# Experiences of text-based synchronous computer-mediated communication in primary care: a qualitative study of registered nurses' experience

**DOI:** 10.3389/frhs.2026.1836878

**Published:** 2026-07-06

**Authors:** Maria Snogren, Sophie Mårtensson, Frida Gradén, Ida Thorsson, Cecilia Åberg

**Affiliations:** 1 School of Health Sciences, University of Skövde, Skövde, Sweden; 2 Närhälsan Töreboda Primary Health Care Center, Töreboda, Sweden; 3 Närhälsan Hentorp Health Care Center, Skövde, Sweden

**Keywords:** consultation, digital communication, person-centered care, primary healthcare, telehealth nursing

## Abstract

**Introduction:**

Digital systems such as synchronous computer-mediated communication have become a complement to traditional healthcare visits in primary care. The aim was to describe district nurses' experiences of digital communication with patients via text-based synchronous computer-mediated communication.

**Methods:**

The study employed a qualitative design, and data were collected through a qualitative questionnaire that included open-ended questions, which were completed by 34 registered nurses working within primary care. The data were analysed using inductive qualitative content analysis. The Consolidated Criteria for Reporting Qualitative Research Elements were considered when conducting the study.

**Results:**

The findings indicate that chat communication presents various challenges for registered nurses, particularly in assessing patients' health status and engaging in caring communication. Digital communication via chat appears most suitable for patient encounters involving uncomplicated and clearly articulatable needs. The findings highlight the need for more knowledge, clearer guidelines, and adapted work routines to ensure that this communication modality supports safe and person-centered care.

**Discussion:**

Clearer guidelines are needed regarding when communication modality form is appropriate and when alternative modalities such as a telephone call or face-to-face contact should be used. Complementary communication pathways must be readily available, and routines should enable early transition to another modality when the patient's needs exceed what written communication can accommodate.

## Introduction

Ongoing population growth is placing increasing pressure on healthcare services globally. Digital solutions offer one approach for creating more efficient and equitable healthcare systems and thereby alleviating some of this pressure. One example of this is the use of digital communication tools such as text-based synchronous computer-mediated communication (text-based SCMC; i.e., text-based communication in real time) as a complement to traditional healthcare visits in primary care (1, 2). The primary care mandate is to provide first-contact, continuous, comprehensive, and coordinated health services to the population, encompassing preventive, diagnostic, therapeutic, and rehabilitative interventions throughout the life course (3). Text-based SCMC enables contact between healthcare professionals (such as district nurses in primary care) and patients as a medium for providing advice and performing assessments. Consultation via text-based SCMC can be used as a replacement for a physical care visit or as a complement and increases accessibility for patients with limited verbal communication skills or patients who prefer written communication for other reasons (4). Although younger people are more positive towards the concept of text-based SCMC as a way of accessing primary care, older people need more support and guidance in its use (2, 5). Text- based SCMC can be seen as person-centred digital care designed around the individual's needs, preferences, and active participation in the care process. Person-centred digital care enhances communication, patient empowerment, and collaboration between patients and healthcare professionals (such as district nurses in primary care) (6). To give person-centred care, three simple routines need to be included (1) establishing a partnership with the patient, (2) Shared decision-making in partnership and (3) documentation in patient records, not only sanctions the value of this information but it also contributes to the continuity and transparency of the partnership (7). Text-based SCMC is associated with the additional shortcoming that non-verbal communication leads to the loss of person-centred care (7). This is problematic because, for example, district nurses can use such non-verbal communication to convey compassion or obtain cues about the patient's state of mind via their body language. Language and communication barriers can also become even more of a problem when text-based SCMC is used for healthcare consultations; the patient may have difficulty describing their symptoms and become anxious or agitated, which would then negatively affect the quality of the consultation (8, 9) and the person-centred care partnership (7). Written language can also increase the risk of misunderstanding given that writing a text often enables less information to be conveyed than in a conversation over the phone or face to face, while the nuances in the language can also be lost. This raises concerns among district nurses that such loss of nuance could lead to inaccuracies in some of their assessments (9). Healthcare professionals, such as district nurses, also vary in their knowledge of how to use digital systems, including text-based SCMC, the application of which was reported to contribute to an increase in administrative tasks and the increased stress of being constantly available (8). It has also been described that text-based SCMC puts important gains in healthcare quality at risk such as person-centred care, when digital systems do not work together, which can lead to frustration and increased workload (2, 5). However, text-based SCMC has been reported by healthcare professionals, such as district nurses, to increase efficiency and improve the ability to offer care appointments at shorter notice, which can be seen as person-centred care. Such increased efficiency contributes to improved accessibility, enabling healthcare providers to spend more time on patients with more complex health problems (10) and improve person centerd care (7). Since district nurses are patients' first contact in primary care, high demands are placed on their ability to perform person-centred care and accurate assessments of each patient's condition without missing important information that could subsequently expose the patient to risk, even when the contact occurs digitally. Given that non-verbal forms of communication, such as tone of voice, facial expressions, and body language, are lost when communication occurs in written form, there is a need to describe the experiences of district nurses working in primary care regarding communicating with patients via text-based SCMC. This study aims, therefore, to describe district nurses' experiences of digital communication with patients by text-based SCMC.

## Methods

### Design

This study employed a qualitative design to describe district nurses' experiences of digital communication with patients by text-based SCMC. Data were collected through a qualitative questionnaire that included open-ended questions and was completed by registered nurses with or without specialist qualifications and working within primary care in the southern part of Sweden. The data were analyzed using inductive qualitative content analysis (11, 12) to systematically interpret district nurses experiences allowing for an in-depth understanding of meanings and patterns within the collected data analysis (11, 12). This study was conducted in accordance with the Consolidated Criteria for Reporting Qualitative Research (COREQ) (13).

### Participants and recruitment

The study used a purposive sampling strategy, as participants were selected based on predefined inclusion criteria relevant to the research aim. Elements of convenience sampling were also present, since recruitment depended on primary care units that agreed to distribute the study invitation. The sample comprised 34 registered nurses with or without specialist qualifications and working within primary care in the Västra Götaland region of Sweden. Participants were included if they were registered nurses with experience of communicating with patients by text-based SCMC and were able to read and understand Swedish. Participants were recruited in January and February 2025 by first providing written and verbal information about the study to the heads of 66 primary care units. Nine of these agreed to help to recruit registered nurses working for their primary care unit, via e-mail. The e-mail sent to the potential participants contained a link to the questionnaire and a letter containing information about the study and its purpose, along with a statement describing that they were considered to be giving their consent to participate by answering the questions. Demographic and professional information on the nurses who agreed to participate is presented in Table 1.

**Table 1:** Demographic/professional information on the participants (*n* = 34).

Demographic/professional information	*n* (%)
Gender	
Female	30 (88.2)
Male	4 (11.8)
Age (years)	
20–30	2 (5.9)
31–40	6 (17.6)
41–50	10 (29.4)
51–60	11 (32.3)
61–65	5 (14.7)
≥66	0 (0.0)
Years in nursing profession	
1–5	3 (8.8)
6–10	6 (17.6)
11–14	3 (8.8)
≥15	22 (64.7)
Specialist qualification	
District nurse	22 (64.7)
Other specialist qualification	3 (8.8)
No specialist qualification	2 (5.9)
Missing	7 (20.6)
Years working in primary care	
<1	3 (8.8)
1–3	10 (29.4)
3–5	6 (17.6)
5–10	11 (32.3)
Missing	4 (11.8)
Number of times using text-based SCMC	
1–5	10 (29.4)
6–10	14 (41.2)
11–15	6 (17.6)
≥16	4 (11.8)

### Data collection

The questionnaire was designed by the authors and contained demographic and professional questions regarding variables including age, sex, and number of years in the nursing profession, and open-ended questions that were designed to shed light on the main issues pursued in this study. The form contained a total of 16 questions, 7 of which concerned the participants' demographic and professional information and 9 of which were open-ended questions. In the response box for each open- ended question, there was no limit on the number of words or characters (see Supplementary Appendix). Prior to its use in this study, the questionnaire was tested on two district nursing students to assess its clarity, relevance, and comprehensibility, and to identify any potential issues with wording, structure, or interpretation prior to full-scale data collection.

The obtained data from the pilot test were not included in the analysis since these two students were colleagues of some of the authors, and the data were not considered representative, given that the informants were aware that their answers would be reviewed and attributed to them personally.

### Data analysis

The responses to the open-ended questions were analyzed as texts using inductive qualitative content analysis (11, 12). This analysis included five steps and was an interactive process that moved back and forth across the steps, not linearly (11, 12). In the first step, all free-text answers were read several times to obtain a comprehensive overview. Text that was determined to be significant to the aim of the research was then extracted and brought together into a single text, constituting the unit of analysis in the second step. In the third step, meaning units related to the purpose of the study were identified and condensed, while preserving the meaning of the text. In the fourth step, the condensed meaning units were abstracted and labelled with a code. The context was taken into account when condensing and coding. Codes that highlighted the meaning of the condensation were created. The codes were compared based on differences and similarities. In the fifth step, the codes were sorted into categories that reflected the data's manifest content. The common content was presented in a main overarching category (11, 12). The analysis resulted in two overarching categories with two or three subcategories each. To boost the robustness of the results, all authors were comparing and discussing the results until a consensus was reached (11, 12). An example of the analysis process is presented in Table 2.

**Table 2:** Example of the analysis process.

Meaning unit	Condensed meaning unit	Code	Subcategory	Category
“It is often difficult to determine how serious the situation is. When you can listen to the patient, you can hear whether they are experiencing breathing difficulties or pain. You can also tell from the patient's voice what a cold sounds like, for example hoarseness, coughing, or whether the patient sounds depressed or tearful.”	Lack of verbal cues makes it difficult to assess symptom severity, physical condition, and emotional state.	Difficulty assessing patients without verbal cues	Challenges in assessing the patient's condition	Challenges in providing holistic and caring communication
“You often have to wait for the patient to respond, ask follow-up questions, and sometimes repeat your questions because the patient does not answer what was asked.”	Delayed or incomplete responses make communication time-consuming and complicate the dialog	Delayed responses hinder communication flow	Barriers in written dialog	
“Even in written communication, you can of course be polite and show goodwill, but it becomes more difficult to convey compassion.”	Written communication limits the ability to convey empathy and compassion.	Difficulty conveying empathy through text	Loss of emotional and personal connection	

### Ethics

According to the Swedish Ethical Review Act (14), no ethical review was required for this study because no personal data were collected for it. However, this work was performed in accordance with the tenets of the Declaration of Helsinki (15), while the obtained data are stored of the Archive Act (16) to ensure the integrity and ethical rigor of the research. The participants received contact information of the authors in case of questions. They were also informed that participation in the study was voluntary, that their answers were anonymous, and that filling in the questionnaire was considered to constitute the granting of consent to participate. The data collection and data analysis were performed with no dependent relationship between the researchers and the participating respondents (16).

## Results

Categories and subcategories based on the registered nurses’ descriptions of their experiences of digital communication with patients via text-based SCMC are presented in Table 3.

**Table 3:** Categories and subcategories based on registered nurses’ experiences of digital communication with patients via text-based SCMC.

Categories	Subcategories
Challenges in providing holistic and caring communication	- Challenges in assessing the patient's condition - Barriers in written dialog - Loss of emotional and personal connection
Enabling efficient and focused communication for less complex healthcare needs	- Preparatory information - Rapid and focused dialog

### Challenges in Providing Holistic and Caring Communication

The findings indicate that text-based SCMC created a tension between efficiency and relational aspects of care. While this mode of communication was perceived as facilitating structured, rapid and focused consultations for less complex care needs, it was also described as limiting registered nurses' ability to conduct holistic assessments and convey empathy in more complex situations. The registered nurses' experiences reflected an ongoing balancing act between maintaining efficiency and preserving caring communication. This category comprised three subcategories: *Challenges in Assessing the Patient’s Condition, Barriers in Written Dialog,* and *Loss of Emotional and Personal Connection.*

#### Challenges in Assessing the Patient’s Condition

Participants described that text-based SCMC limited their ability to form a comprehensive understanding of the patient’s overall health status. This was particularly evident when patients presented vague, nonspecific or complex symptoms. The lack of auditory and visual cues was perceived as reducing registered nurses' ability to evaluate symptom severity and interpret the patient's emotional or physical condition.

It’s difficult to grasp how serious the situation is. When you can listen to the patient, you can hear whether they have breathing difficulties or are in pain.

Registered nurses relied heavily on patients' written descriptions and on their own ability to formulate sufficiently precise follow-up questions. This created uncertainty about whether important information might be overlooked, particularly when communication occurred through relatives or guardians.

#### Barriers in Written Dialog

Participants described that text-based SCMC altered the flow and dynamics of the healthcare dialog. Delayed patient responses, unanswered questions, and writing- and language-related difficulties were perceived as obstacles that complicated consultations and prolonged communication processes. Registered nurses emphasized the importance of formulating messages carefully and precisely in order to avoid misunderstandings.

You have to be careful about how you phrase things so there are no ambiguities.

Written communication required greater deliberation and linguistic precision compared with spoken interaction. Misunderstandings could not easily be clarified and could negatively affect the continued dialog.

#### Loss of Emotional and Personal Connection

Participants described that text-based SCMC limited opportunities to establish emotional and personal connection with patients. Although registered nurses attempted to convey attentiveness, engagement and empathy through written language, many experienced difficulties in expressing compassion without verbal cues such as tone of voice.

Even in written language, you can of course be polite and show goodwill, but it becomes harder to show compassion.

To compensate for the lack of direct interpersonal interaction, participants described using affirming phrases and repeated expressions intended to demonstrate active listening and support. For certain health problems, *chat*-based communication was perceived as insufficient to provide the support, for example, in cases of mental health concerns. In such cases, registered nurses commonly preferred to switch to telephone communication.

### Enabling Efficient and Focused Communication for Less Complex Healthcare Needs

This category describes that text-based SCMC is perceived as effective when patients seek help or advice for less complex healthcare needs. The format supported focused information exchange and rapid assessment when healthcare concerns were clearly defined. The subcategories *Preparatory Information* and *Rapid and Focused Dialog,* of which this category comprises, are described in more detail below.

#### Preparatory Information

Participants emphasized that preparatory information provided through structured questionnaires before the consultation improved the condition for efficient communication. Access to the patient's reason for seeking care beforehand enabled registered nurses to prepare relevant follow-up questions and approach the consultation in a more focused and structured way.

The patient is guided in what is important for us to know, and a clear focus for the conversation is created.

The questionnaires functioned not only as information-gathering tools but also as support for patients in identifying and communicating relevant aspects of their health concerns. This contributed to more direct consultations and facilitated quicker assessments.

#### Rapid and Focused Dialog

For less complex healthcare needs, participants perceived text-based SCMC as enabling concise and goal-oriented communication. Patients were described as having an opportunity to reflect on their symptoms and formulate their concerns in writing before responding, which often resulted in focused and clear information exchange. Participants also described advantages related to documentations as the written format allowed the registered nurses to reproduce the patient's own expressions accurately.

I can reproduce exactly what the patient has expressed in text, not what I’ve heard.

The written format reduced the need for interpretation or reformulation and created a clear record of the interaction.

## Discussion

The findings of this study indicate that text-based SCMC presents various challenges for registered nurses, particularly in assessing patients' health status and engaging in caring communication. For example, the absence of non-verbal cues makes it difficult to form a comprehensive understanding of the patient's condition, which aligns with the findings of Entezarjou, Sjöbeck (17), who note that digital text-based encounters often lack essential contextual information such as tone of voice and body language. As a result, text-based SCMC interactions frequently need to be supplemented with telephone calls to obtain sufficient detail and be person-centred, especially in cases involving complex medical conditions. Farr, Banks (18) similarly assert that critical nuances may be lost in healthcare consultations in which communication between patient and healthcare professional occurs in written form, increasing the risk of misinterpretation and inadequate clinical assessment.

The results of this work also highlight practical barriers associated with the use of text-based SCMC for healthcare consultations, such as delayed responses and patients’ varying literacy skills, which further complicate the dialogue. The delayed responses also and affects the degree of the ability to deliver person-centred care. These challenges resemble those described in telephone triage, where nurses must rapidly establish a rapport and make accurate assessments in a limited time despite potentially lacking familiarity with the patient (19). Effective communication skills thus remain essential, as emphasized in national competency guidelines for district nurses from the Swedish Nurses' Association (Svensk Sjuksköterskeförening) (20).

In the present study, establishing emotional connection and demonstrating empathy were perceived as more difficult in text-based SCMC, particularly when supporting patients with mental health concerns. This is consistent with the findings of Ekman, Swedberg (7), who stress that caring communication requires an understanding of the patient's lived experience to deliver person-centred care. The reduced ability to convey empathy and interpret subtle cues may hinder the development of a trusting nurse–patient relationship, a key component of effective and person-centred care according to Wiechula, Conroy (21). Previous research by Cajander, Larusdottir (22) also shows that the loss of linguistic nuance in text-based communication increases the risk of misunderstandings, as confirmed in the present study.

The findings of the present study must also be considered in relation to the ongoing transition toward person-centred and accessible primary care within the Swedish “Nära vård” framework (equitable, accessible, and person-centered care) (23). While text-based SCMC can support person-centered care in cases involving simple, clearly articulatable needs facilitated by structured pre-*chat* questionnaires, it appears less suitable for complex situations requiring holistic assessment. This echoes the concerns raised by Entezarjou, Sjöbeck (17) and Farr, Banks (18), who note that text-based communication is particularly limited when non-verbal information is essential for clinical judgment. As such, the development of an individualized care plan, for example, may be difficult when based solely on text-based SCMC. A scoping review by Leonardsen, Bååth (6) also stated also in line with the study result that digitalisation, such as text- based SCMC increase the accessibility of services, patients' ability for self-management and healthcare personnel's situation awareness. Leonardsen, Bååth (6) also in line with the result indicate that digitalisation in primary health care may be at odds with person-centred care, based on the possibility to enhance communication, patient empowerment, and collaboration between patients and healthcare professionals in primary care.

To ensure that person-centered care is provided even when text-based SCMC is used for healthcare consultations, clearer guidelines and adapted workflows are needed, along with training that supports nurses in navigating the limitations of written communication. At the same time, the results show that *chatting* can enhance accessibility and efficiency for straightforward medical cases, consistent with the findings of Eldh, Sverker (24) who describe how digital systems streamline triage and allow patients to contact healthcare professionals at their convenience. However, for complex or emotionally sensitive concerns, especially those involving mental health issues, verbal communication, preferably in person, remains crucial.

## Conclusion

This study highlights that while text-based digital communication, such as text-based SCMC offers efficiency for straightforward clinical tasks, it presents critical limitations for complex healthcare needs requiring holistic assessment and emotional attunement.

The absence of non-verbal cues with text-based SCMC and personal connection directly obstructs nurses in delivering safe, person-centred care, a fundamental tenet of both their professional role and the broader transition toward integrated primary care. Text-based SCMC can be concluded to be utilised as a complementary modality rather than a replacement for more nuanced interpersonal interactions, ensuring the depth of dialogue necessary to maintain person-centred care quality and patient safety in digitalised healthcare.

### Conclusions and clinical implications

To ensure that text-based SCMC supports safe, high-quality, and person-centered care, clearer guidelines at an organisational level are needed regarding when this communication form is appropriate and when alternative modalities such as a telephone call or face-to-face contact should be used. Complementary communication pathways must be readily available, and routines should enable early transition to another modality when the patient's needs exceed what written communication can accommodate. Strengthening nurses' competence in written communication is also essential, particularly in recognizing when text-based SCMC is insufficient for clinical assessment or supportive dialog. Strengthening nurses' competence in written communication in digital triage is also important for strengthening the person-centred benefit of text-based SCMC.

At the organizational level, both healthcare providers and patients should receive clear information about the intended purpose and limitations of text-based SCMC. Establishing structured routines for when and how to shift to another communication channel can enhance patient safety and support person-centered care. Digital tools should be designed to support, not replace, the human relationship that underpins effective nursing practice.

As digital solutions continue to play an increasingly central role in sustainable healthcare development, further research on text-based SCMC is warranted. Future studies should include larger and more diverse samples, explore regional differences, and examine patient perspectives to provide a more comprehensive understanding of text-based SCMC as a communication modality in healthcare. Such knowledge is essential for guiding the continued development of digital communication in primary care and ensuring that it aligns with the principles of person-centered and safe care.

### Strengths and limitations of the study

This study's main strength is that it explores a rarely studied topic: district nurses' experiences of digital communication with patients via text-based SCMC. The results broaden our understanding of when text-based SCMC is effective in primary care and when its limitations obstruct accurate assessment, caring dialog, and person-centered practice.

Another strength of this work is the reliability of the findings, as supported by inclusion of all of the authors in reflective discussions and analysis of the data. Authenticity was secured by including original quotations from the qualitative responses in the questionnaire in presenting the results. Questionnaires were chosen because they enabled data collection from participants across multiple primary care units in a flexible and time-efficient manner. However, unlike interviews, questionnaires did not allow for probing or clarification of responses, which may have limited the depth of the data collected. Content validity was ensured by developing the questionnaire based on existing literature and the study's aim, and by reviewing it for relevance, clarity, and coverage of the intended construct, often with input from experts in the field or the target population. This study's trustworthiness was evaluated using qualitative rigour criteria, including credibility, dependability, confirmability, and transferability. Credibility was supported through systematic data analysis and transparent interpretation, while dependability and confirmability were enhanced by maintaining a consistent analytical process and reflexive documentation to minimize researcher bias; transferability was addressed by providing rich contextual descriptions to enable readers to assess the applicability of the findings to other settings. Credibility and reliability were also secured by including all of the authors in the coding, analysis and interpretation of the data. The study also comprised a number of registered and specialist nurses with different levels of work experience to obtain a purposive sample and to ensure the validity and transferability of the findings (25). The sample was seen as appropriate when performing this type of study, which strengthens credibility. One limitation may be that all participants were from Sweden, and the sample included a majority of females. Including more participants could have presented the results in more depth and differences. On the other hand, data saturation was reached in the collected data, justifying the number of participants. Missing participant data were limited to demographic variables and did not affect the completeness of the qualitative responses included in the analysis. However, the demographic imbalance within the sample, such as differences in gender, age, professional experience, or specialist qualifications, may limit the transferability of the findings, as the experiences represented may not fully reflect the perspectives of all registered nurses working in primary care settings.

## Data Availability

The raw data supporting the conclusions of this article will be made available by the authors, without undue reservation.
