# Supplementary material for: Experiences of text-based synchronous computer-mediated communication in primary care: a qualitative study of registered nurses' experience
Source: Front Health Serv. 2026 Jul 6;6:1836878. doi: 10.3389/frhs.2026.1836878 (PMC13381459; doi:10.3389/frhs.2026.1836878)
Supplement: Supplementary file 1 [file Supplementaryfile1.docx]

**Appendix**: Questionnaire with free-text answers about district/nurses' experiences of digital communication with patients via text-based synchronous computer-mediated communication (text-based SCMC).

| **Background questions:** | | | | | | |
| --- | --- | --- | --- | --- | --- | --- |
| 1 | How old are you in years? | | | | | |
|  | 20-30 | 31-40 | 41-50 | 51-60 | 61-65 | 65< |
| 2 | What gender do you identify with? | | | | | |
|  | women | men | other |  |  |  |
| 3 | How many years have you worked as a registered nurse? | | | | | |
|  | 1-5 | 6-10 | 11-14 | 15< |  |  |
| 4 | How many years have you worked in primary care? | | | | | |
|  | 1-3 | 3-5 | 5-10 | 10< |  |  |
| 5 | Number of carries through of text-based SCMC? | | | | | |
|  | 1-5 | 6-10 | 11-15 | 16< |  |  |
| 6 | Do you have any specialist training? | | | | | |
|  | Yes | No |  |  |  |  |
|  | If yes, which? | | | | | |
|  |  | | | | | |
|  | If yes, what year did you get your specialist degree? | | | | | |
|  |  | | | | | |
| 7 | Which primary care centre do you work at? | | | | | |
|  |  | | | | | |
| **Please answer the following questions by giving examples of situations. There is no maximum word limit.** | | | | | | |
| 8 | How do you experience communicating with patients via text-based SCMC compared to over the phone? | | | | | |
|  |  | | | | | |
| 9 | What benefits do you experience from communicating with patients via text-based SCMC? | | | | | |
|  |  | | | | | |
| 10 | What difficulties do you experience communicating with patients via text-based SCMC? | | | | | |
|  |  | | | | | |
| 11 | How do you feel that the conversation process (opening, listening, analysing, motivating and closing) differs in communication via text-based SCMC compared to telephone? | | | | | |
|  |  | | | | | |
| 12 | How do you feel that your way of communicating via text-based SCMC creates a feeling that you are present and listening when body language and voice cannot be used? Please provide concrete examples. | | | | | |
|  |  | | | | | |
| 13 | How do you feel that the patient can convey their message in communication via text-based SCMC? | | | | | |
|  |  | | | | | |
| 14 | How do you experience the documentation in relation to text-based SCMC? | | | | | |
|  |  | | | | | |
| 15 | Is there anything you would like to change or improve with the text-based SCMC? | | | | | |
|  |  | | | | | |
| 16 | Is there anything else you would like to convey regarding communication via text-based SCMC? | | | | | |
|  |  | | | | | |
